# Auto-amputated Ovarian Cyst with Compression Sequelae: A Case Report

**Published:** 2012-10-01

**Authors:** Bilal Mirza

**Affiliations:** Department of Pediatric Surgery, The Children's Hospital and the Institute of Child Health Lahore, Pakistan.

**Keywords:** Ovarian cyst, Fallopian tubal atresia, Ileal stenosis

## Abstract

Ovarian cysts contribute a major share of cystic lesions in fetal life. Quite often, these cysts are benign and resolve spontaneously. Occasionally, these cysts can twist, resulting in ovarian loss. We report a case of auto-amputated ovarian cyst presetting with intestinal obstruction.

## INTRODUCTION

Majority of cystic lesions in the female fetus, identified on antenatal scans, are ovarian cysts; of which common are follicular and luteal cysts. These cysts are often benign and tend to resolve spontaneously in intrauterine life or within few months after the child has born [1].

The bigger cysts (more than 4cm) may complicate; sinister being the ovarian loss due to torsion, auto-amputation, and hemorrhage inside the cyst [2, 3]. Complications as a result of compression sequelae such as hydronephrosis, intestinal obstruction, polyhydramnios etc. are seldom reported [3, 4]. We report a case of auto-amputated ovarian cyst in a patient presenting with small bowel obstruction. The cyst caused compression sequels on ileum and fallopian tube.

## CASE REPORT

A 28-day-old female baby was presented to the surgical emergency of our institution with abdominal distension, bilious vomiting, constipation and reluctance to feed for 3 days. Few antenatal scans available gave no abnormality in the fetus. The baby was a product of spontaneous vaginal delivery at term. The baby initially tolerated feeds during the first week of life, but developed vomiting thereafter. The frequency of vomiting had increased to 4-5 times a day over three weeks. Three days prior to the presentation, she developed abdominal distension, bilious vomiting after every feed and constipation. 

On clinical examination, abdomen was distended with visible gut loops. On palpation, no mass lesion was present. The bowel sounds were absent and digital rectal examination revealed an empty rectum. Provisional diagnosis of intestinal obstruction was made and patient was investigated. Patient was initially resuscitated with intravenous fluids. Naso-gastric tube was passed and antibiotics started. Abdominal radiograph performed showed multiple air fluid levels. Ultrasound showed massive gaseous distension of the intestinal loops obscuring other viscera. Laboratory tests were within normal limits. The patient underwent exploratory laparotomy which showed a stenosis of mid-ileum causing small bowel obstruction (Fig. 1). A dark brown colored cyst was present in the pelvis having a grayish nodular thickening on one side (Fig. 2). The cyst was freely floating in the peritoneal cavity; not attached to the surrounding structures. 

**Figure:**
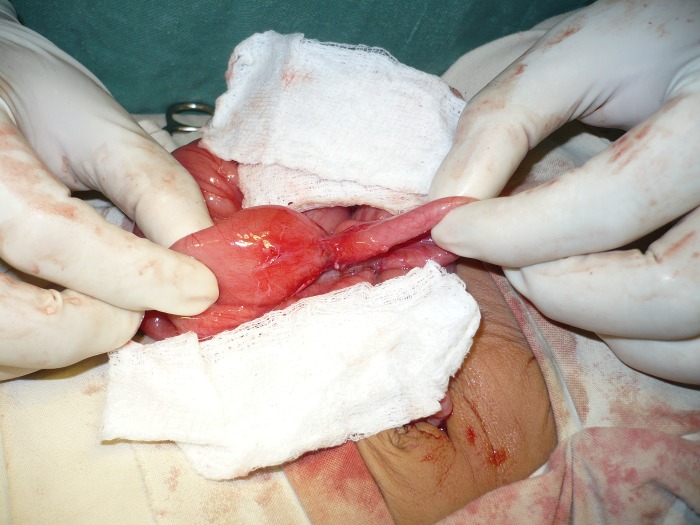
Figure 1: Ileal stenosis.

**Figure:**
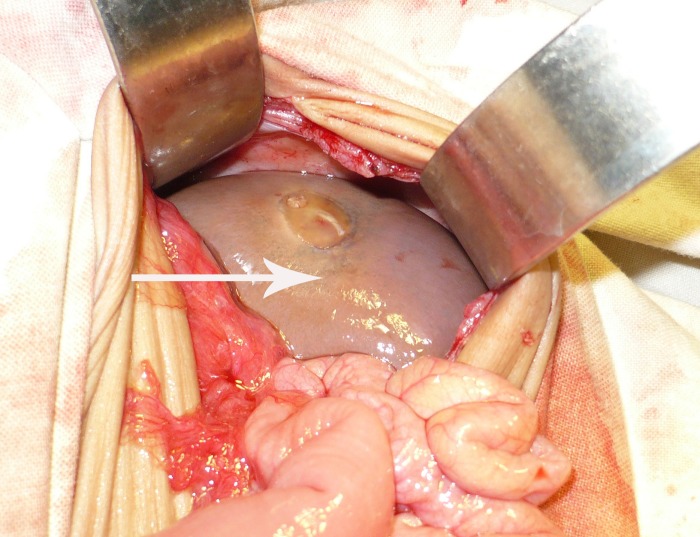
Figure 2: A grayish cyst in the abdomen.

Further exploration revealed absence of left sided ovary. Moreover, left sided fimbrial end of fallopian tube was freely floating in the abdomen. Some fibers of connective tissue were present between the fimbrial end and the uterine end of the fallopian tube and the portion in between was absent (Fig. 3). Right sided ovary and fallopian tube were normal. 

**Figure:**
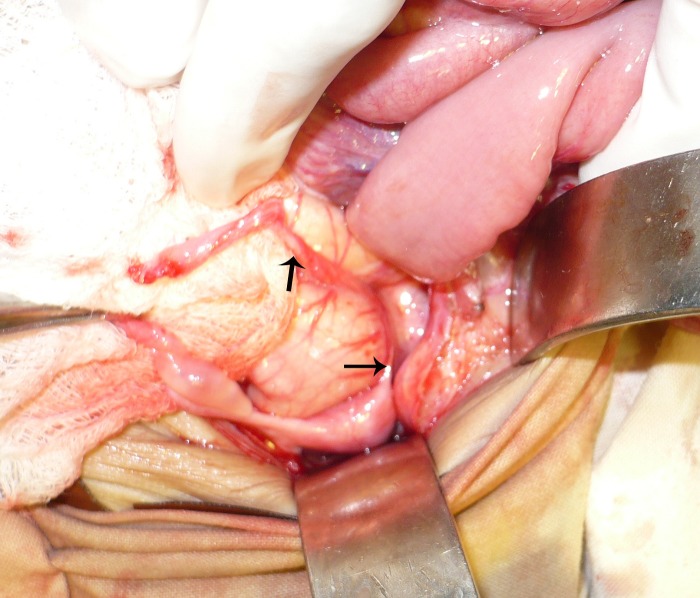
Figure 3: The portion of fallopian tube marked with two arrows was atretic.

Resection and ileo-ileal end to end anastomosis was performed for ileal stenosis. Cyst was eviscerated and sent for histopathology which revealed a benign ovarian cyst with hemorrhagic fluid inside. Post operative recovery was uneventful. Patient visited us for 6 months and then never returned.

## DISCUSSION

Ovarian cysts are categorized as simple and complex cysts based on sonographic findings. Simple cysts have well defined walls and are anechoic, while, complex cysts have debris fluid levels, septae or retracting clot within the cyst [1-3]. The exact etiology of fetal ovarian cyst is not known; however, it is believed the raised levels of chorionic gonadotrophins play a role. The usual course of these cysts (less than 4cm) is spontaneous resolution in intrauterine life or within few months of the birth on account of marked reduction in maternal hormonal stimulation [3, 4]. 

Bigger cysts (more than 4cm) are prone to various complications like torsion leading to ovarian loss, hemorrhage and pressure sequelae. To prevent such dreadful complications, ultrasound guided in-utero aspiration of bigger cysts is advocated. This not only reduces the size of the cysts but also the chances of ovarian loss; nevertheless, multiple aspirations and an expert sonologist are the prerequisites [1, 2]. Intrauterine ultrasound guided puncture of the cyst is also advised to reduce the morbidity [2]. In case of cyst torsion, auto-amputation, suspicion of cyst rupture, or hemorrhage inside the cyst, surgical intervention becomes necessary [3, 4]. Ovarian cyst can cause compression effects on surrounding structures which may result in polyhydramnios, hydronephrosis, intestinal obstruction, and perforation [3]. In our case, the cyst had compression effects on the intestine and the fallopian tube. Zampieri et al reported a case of antenatal ovarian torsion and ipsilateral complete tubal atresia in 2009. In their case, they proposed the combined tubo-ovarian torsion as etiology for their findings [7]. Our case is unique because the patient presented with signs of intestinal obstruction and the auto-amputated ovarian cyst was an incidental finding. One mechanism that can explain the ipsilateral tubal atresia is combined tubo-ovarian torsion. However, presence of ileal stenosis and atresia of only central portion of fallopian tube are more plausibly explained by long term compression effect of the auto-amputated ovarian cyst on the ileum and fallopian tube. 

It can be concluded that antenatal diagnosis and close surveillance is necessary in a case of ovarian cyst to prevent ovarian loss as happened in our case. Auto-amputated ovarian cyst may present with symptoms secondary to its compression effect.

## Footnotes

**Source of Support:** Nil

**Conflict of Interest:** The author is a Joint Editor of the journal. But he did not take part in the evaluation or decision making of this manuscript. The manuscript has been independently handled by two other editors.
